# The ecological dynamics of trumpet improvisation

**DOI:** 10.1007/s10339-023-01159-9

**Published:** 2023-09-22

**Authors:** Miles Rooney

**Affiliations:** Faculty of Fine Arts and Music, University of Melbourne, Melbourne, Australia

**Keywords:** Improvisation, Ecological psychology, Dynamic systems theory, Affordances, Embodiment, 4E cognition

## Abstract

The nature of music improvisation continues to provide an interesting showcase of the multifaceted and skilful ways we engage with and act within our environments. Improvising musicians are somehow able to generate musical material in real time that adaptively navigates musical situations. In this article I explore the broader aspects of improvised activity—such as our bodily interactions with the instrument and environment—as they relate to improvised music-making. I do so by drawing upon principles from the embodied cognitive sciences, namely ecological and dynamical systems approaches. Firstly, I introduce the concept of affordances to illustrate the bidirectional relationship between improvisor and environment. I then take a dynamical view, exploring the ways that a trumpet player coordinates their body with their instrument and engages with trumpet affordances in order to navigate musical situations. I continue this dynamical view, taking the improviser to be an adaptive system whose behaviours are self-organised responses to a set of constraints. To conclude, I situate my research within the wider 4E approach. I advocate that ‘E’ approaches, which take seriously the role of the body–instrument–environment relationship, provide an insightful perspective on the nature of improvisation.

## Introduction

Improvisation is a term that can be difficult to define. Commonly it refers to a form of unscripted activity, where an agent is able to come up with patterns of behaviour in the moment. For example, improvising musicians are able to produce complex melodic, rhythmic, and textural material in the moment, while simultaneously responding adaptively to situations. Two well-known models of musical improvisation are provided by Pressing (1988) and Johnson-Laird (2002). The former is a reductionist view that improvisation involves learned event clusters that are ordered through *associative* or *interrupt generation*. The latter, in contrast, suggests that improvisers learn and apply different rules or algorithms. Norgaard (2011) proposes a combination where improvisation is, at different times and depending on the situation, one or the other. What these models have in common is their emphasis on pitch or melodic generation and their commitment to in-the-head processing theories of cognition. As an alternative, this paper takes a broader view of improvisation, one that is continuous with the kinds of skilful and embodied engagement intrinsic to everyday life. When we perform intentional or goal-related activities, the environments we move through are not static, nor are they necessarily accommodating. A broader sense of improvisation describes our sensitivity to features of the dynamic and ever-changing environments we inhabit, and the adaptive behaviours we develop to skilfully cope with environmental pressures (Krueger and Salice 2021). Improvisation involves ongoing interactions between the body, environment, technologies, and social norms–it is a multidimensional skilled activity that is an inherent part of our lives. To capture the dynamic relationship between body and environment in improvisation, I utilise principles from ecological psychology and dynamical systems theory—approaches well acquainted with the idea of fluid skilled action.

The first section of this paper will introduce the ecological approach and the concept of *affordances* or possibilities for action. The ecological approach considers perception in terms of how one can interact with their environment, thus usefully establishing a framework that acknowledges the reciprocal relationship between an agent (in this case improviser), object (instrument), and environment. In the second section, I discuss the dynamic relationship between trumpet and trumpeter, examining how the improviser engages with available affordances to navigate musical situations. This enquiry into the musician–instrument–environment relationship is framed by theoretical work from the cognitive sciences, literature surrounding brass playing, and my personal insight as a professional improviser and trumpet player. I have also attempted to present my discussions of trumpet playing in a way that is intuitive and accessible to non-trumpet players. Continuing this dynamical perspective, the third section considers the improviser as an adaptive self-organising system under a set of control parameters or *constraints*. In the final section I briefly look at how the addition of other Es (embodied, embedded, enactive, and extended) may develop this approach going forward.

## Affordances in improvisation

The term affordance was used, most notably, by James Gibson (1979) in The Ecological Approach to Visual Perception. Affordances play a pivotal role in the shift away from dominant cognitive theories that focus on in-the-head processing, instead placing emphasis on the direct role played by the environment. Gibson believed that the world contained structured meaningful information that could be directly perceived by the observer. This theory extends upon Gestalt psychology’s notion of valences–that the value of objects in the environment is immediately apparent and so can invite interaction of various kinds. Gibson states that “The post box invites the mailing of a letter, the handle wants to be grasped, and things tell us what to do with them” (p. 130). When the observer directly perceives the environment, they perceive the kinds of action and interaction possibilities *afforded* to them. Gibson writes “Affordances of the environment are what it offers the animal, what it provides or furnishes, either for good or ill” (p. 119). Affordances also exhibit a bidirectionality. That is, they are not subjective or objective but instead an emergent property of the two. Gibson is quite explicit about this:

An affordance is neither an objective property nor a subjective property; or it is both if you like. An affordance cuts across the dichotomy of subjective-objective and helps us to understand its inadequacy. It is equally a fact of the environment and a fact of behaviour. It is both physical and psychical, yet neither. An affordance points both ways, to the environment and to the observer (p. 129).

Affordances are out there in the environment, but they are not abstract qualities. They are the world perceived in relation to aspects of the agent, such as their physiology and abilities. This has been an important insight into the field of skill development, particularly in sports. Chow et al. (2016) suggests that:

Affordances are available in every performance environment to be used to regulate behaviours. Affordances should not be considered as entities that are perceived but rather as functional relationships formed between an individual performer and a performance environment. This definition emphasises the functional, rather than structural, properties of a performance environment (i.e. what an object, surface or another individual offers an athlete in terms of opportunities for actions). (p. 30)

What we might gather from this is that affordances denote the interactive aspects of the environment: we perceive in terms of what we can do. With this in mind, what are the affordances available to an improvising musician? Firstly, the instrument itself affords several action possibilities. On a trumpet, the mouthpiece placed into the lead pipe affords a tone to an agent with the ability to form an embouchure. Notice that the tone is not intrinsic to just the trumpet or the trumpet player but is an emergent relationship between the two.

The instrument provides a landscape of affordances, and the types of affordances available solicit certain kinds of interactions (due to the relation between the structure of the trumpet and the trumpeter’s physiology) (Rietveld and Kiverstein 2014). According to Rietveld et al. (2018), solicitations are “the affordances that show up as relevant to a situated individual, and generate bodily states of action readiness” (p. 11). Furthermore, we skilfully engage with multiple relevant affordances simultaneously. For an improvisor, this may be in respect to not only the physical relationship between body and instrument but also the improvisor’s creative goals, aesthetic preferences, and performance situation. While an object or situation may afford a multitude of action possibilities, an improvisor is “drawn to affordances that they care about and are able to act on” (p. 12). That is, the affordances relevant to performing the appropriate actions will have a greater inviting character. “These solicitations “stand out” as relevant (against the background of other affordances in the situation)” (p. 15). A musician performing a composed work will be drawn to affordances related to successfully performing that work. However, an improvisor with more open-ended performance goals may experience a different set of solicitations. For example, many improvisors are highly sensitive to the soliciting affordances provided by the unique construction of various instruments. This is sometimes referred to as instrumental idiomaticity (De Souza 2017; Huron and Berec 2009). That is, the relationship between the structure of the instrument and the structure of the body encourages certain kinds of interactions. Instruments with a regular spacing of intervals and frets that are consistent semitones (like the guitar) can solicit or encourage the use of chromaticism. The guitarist can maintain the same hand shapes and simply move side to side or up and down. Changing the tuning to irregular intervals (like an open D tuning) instead makes it desirable to perform diatonically within the key of D. A guitarist could play chromatically in an open D tuning if the task specifically prescribed it; however, it is important to note the difference between what is specifically prescribed and what actions the instrument invites. Historically these idiomatic features have, over time, shaped the developments in musical genres and playing styles (De Souza 2017; Rockwell 2009). In later sections I will look at idiosyncratic features of the trumpet such as the mouthpiece, partial series, and valves. These properties afford and invite interactions and ways of improvising distinct from other instruments such as the guitar.

Although certain affordances will stand out to improvisors, they need not always respond to these solicitations. At times improvisors will engage with affordances for functional reasons, and sometimes for aesthetic or creative ones–and these affordances may be less obvious choices or even hidden. For many musicians, the preparatory phases of their practice are often spent exploring and discovering these less obvious or hidden affordances to innovate novel forms of timbre, expression, rhythm, and melody. For example, a trumpet player can manipulate the embouchure to activate the trumpet in such a way that it produces either multiple clear tones or adds a type of distortion to the sound. This is commonly referred to as a split-tone.^1^ Producing a split-tone deviates quite dramatically from conventional trumpet playing and can be unintuitive and difficult to perform consistently. Improvisors developing this technique must spend considerable time searching for and learning to access these “split-tone” affordances.

## Perceiving affordances

The ecological approach suggests that the way we perceive and interact with affordances is shaped by intention, attention, and calibration (Jacobs and Michaels 2007). These perceptual systems act to direct and differentiate the information in the environment being perceived by the observer. As previously mentioned, an object may afford a number of possible interactions, but what is of importance to the observer is the affordances relevant to performance tasks. One’s *intention* sets the goal or task needed to be completed indicating what the affordances should be relevant to. For example, the types of trumpet affordances perceived differ greatly depending on if I intend to play the trumpet or clean it. Similarly, affordances may differ depending on if the performance involves free improvisation, chord changes, genre-specific improvisational language, or a composed work. Moreover, I may intend to fit neatly within these contexts, or I may want to go against them, enacting new worlds of salience. *Attention* refers to our sensitivity to relevant and beneficial affordances. Our capacity to skilfully interact with the world relies on the ability to perceive affordances that help us complete our intended tasks. However, over time our attention to relevant affordances and our bodily dimensions and action capacities develop and change. A constant process of *calibration* is required to maintain tight integration between perception and action (Araujo et al. 2009). For example, while a trumpet remains relatively unchanging the trumpeter can feel very different day to day. Things you could play one day may not come so easily the next. Often, a warmup involves a process of calibrating the affordances perceived with the abilities of the body on that day. A final point on the perception of affordances is that while affordances provide perceptual information that shapes behaviour, behaviour (as the agent moves through the environment) changes the array of available affordances (Di Paolo et al. 2017). This dynamical view of affordances is also introduced by Chemero (2009) as affordances 2.0. He suggests that affordances “causally interact in real-time and are causally dependent on one another” (p. 151).

To summarise, an expert improvisor is attuned (through a history of interactions with the environment) to affordances relevant to their task domain. They can also adapt these affordances to suit the contingencies of a given situation. The affordances significant to engaging with the trumpet within an improvised environment stand out to the improvisor. And the instrument becomes an integral part of the domain of musical meaning making, which includes musician, instrument, and environment (the social, acoustic, and material space) as an extended evolving system. The trumpeter’s attention is on functional ways of engaging with trumpet affordances, and as the agent–environment relationship is continually changing, they are constantly calibrating and adapting to maintain or enact new musical relationships.

## Sociocultural environments

In more recent years, the role of affordances has been extended further to account for not only our physical environments but the complex sociocultural environments and practices we are embedded in. The complexity of the human way of life reveals a further layer to the previous definitions, adding a socio-normative aspect to the perception of and interaction with affordances. Not only to we act in response to physical features of the environment, we must also engage with and act appropriately within certain social norms (Rietveld 2008; Rietveld and Kiverstein 2014). McLean (2018) illustrates this in his development of a solo drum set practice. Although his practice is largely focussed on exploring patterns of behaviour based on his sensorimotor interactions with the drum kit, he also situates his research within a community of musicians referred to as antipodean improvisors. He outlines the community’s antecedents and current members as well as his relationship with them and their influence on his music practice and research. This community is a small group of improvising musicians within Australia that are defined under a variety of criteria. Although he does not explicitly use the term affordance, by specifying the environments his embodied practice is embedded within, Mclean demonstrates the interwoven physical and social nature of his aesthetic selection and development process. His patterns of behaviour are not only afforded to him by the drum set but by the social community he is embedded in.

## Coordinating body and trumpet

So far, we have seen that the instrument and environment present us with a range of opportunities for action with varying degrees of solicitation. In this section, I illustrate some ways an improvisor coordinates their body with instrumental affordances, specifically within the context of trumpet playing. I do so by detailing the processes of sound production, pitch changes, articulation, and use of the valves. These sensorimotor schemes play an important role in the generation of melodic, rhythmic, and timbral material. I draw upon not only the surrounding literature, but also my observations and experiences as a practitioner-researcher, professional trumpeter, and improviser.

Producing even just a single note on the trumpet involves a complex coordination of the trumpeter’s physiology (respiratory muscles, throat, tongue, embouchure, lips, hands, and posture) and the material and mechanical aspects of the instrument (valves, mouthpiece, bore size, bell dimensions, and type of metal). This type of behaviour is referred to as synergy—that is, the temporary grouping and unification of elements within a system to produce emergent outcomes (Kelso 2012). The initial source of energy (air) comes from the respiratory muscles. Bouhuys (1969) refers to the chest as a kind of “elastic bellows” (p. 1200), it has a natural resting position it returns to. If we inspire to full capacity and then relax the inspiratory muscles, the chest returns to the resting position and the air is expired. The inspiratory muscles can also act as a kind of brake, managing the decrease in chest volume as it returns to its resting position and controlling the rate that the air is expired. The expiratory muscles can then be employed to maintain a constant expiration beyond the resting position. Furthermore, the expiratory muscles can help to produce greater pressure beyond the natural elasticity of the chest. Bouhuys states that: “To accomplish a simple action like breathing out against a constant pressure requires a complex motor act which involves precise regulation of the state of contraction of both inspiratory muscles and expiratory muscles” (p. 1201). The air column produced by the respiratory muscles forms a positive pressure behind the closed lips, pushing the lips open and producing vertical and horizontal motions. As the air flows out, the pressure changes, and the lips close. This set of events repeats as a cycle sending pulses of high-pressure air into the instrument. The auto-oscillation of the lips (repeated open–close cycle) collaborates with the natural resonances of the instrument to produce notes in the partial series (also referred to as harmonic series) (Boutin et al. 2015; Wolfe et al. 2015).

**Fig. 1:**
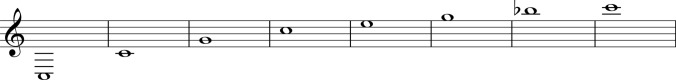
Bb trumpet partial series

The fundamental pitch and the harmonic series of the instrument are dependent on the length of the pipe. The valves provide access to more tubing, allowing transposition of the available partial series when used in combination. The trumpet’s fundamental pitch with no valves depressed is C (or concert Bb), with each of the six subsequent valve combinations lowering this by one semitone. Using the valves, the trumpet player has the opportunity to not only move between partials—as is done on a natural trumpet—but also within partials (Fig. 1).

The movement between partials is a result of airspeed being manipulated by the aperture of the lips and by the tongue position. This is in relation to the air pressure initiated by the respiratory muscles. By adjusting the size of the aperture, the trumpet player produces a faster airstream and a higher frequency (pitch) or a slower airstream and a lower frequency. The amplitude of the tone produced by the trumpet correlates to the volume of air being accelerated through the instrument. By opening the aperture wider, a greater volume of air may travel into the trumpet. A consequence, however, is the way that this affects airspeed, meaning there is greater reliance on the initial respiratory processes to maintain enough pressure to hold and change pitch. Consider a garden hose: if one puts their thumb over the nozzle, the output of water is faster and at a higher pressure. This is opposed to increasing the volume of water output by turning the tap. Thus, to interact with the partial affordances, the trumpet player must coordinate the air generator (inspiratory and expiratory muscles) and air manipulators (tongue, aperture, and embouchure). They must make synergetic adjustments to control and maintain air pressure, speed, and volume in order to control pitch changes, amplitude, and timbre.

## Taxonomy of articulations

While improvising, the trumpet player draws on a wide repertoire of articulations. These articulations can play both an aesthetic and functional role when navigating musical situations. The movement between partials, produced only by a change in aperture and tongue position, is referred to as a *lip slur*. If this movement is initially articulated by the tongue it is referred to as a *tweet*. A *bugle* is a movement between partials where both partials are articulated by the tongue. Both the bugle and the tweet make use of the momentum from the initially articulated note to move between partials. Articulations are the different onsets of notes produced by the trumpeter. A basic example of this is the breath attack where air generated by the trumpet player produces a vibration in the lips $\to$ mouthpiece $\to$ trumpet producing a tone. Building from this foundation, the *single tongue* is an articulation type that utilises the interactions between the air generated by the trumpeter and the tongue. The tongue blocks the flow of air into the trumpet creating compression in the mouth. When released, the compressed air produces a note with a faster and harder onset than the breath attack. Producing a breath attack requires full use of the respiratory muscles making it a cumbersome method of articulation. The tongue instead makes use of air stored in the mouth, allowing for greater agility and a more nuanced range of attacks. For example, varying levels of compression can produce a range of onset attack types like tah, dah, and dat sounds (Wolfe et al. 2015). The *slur* refers to movements between pitches produced by changes in valve combinations only, without the use of the tongue. The trumpeter will often use tongued and slurred articulations in combination–referred to as back-tonguing. Finally, the ghost tongue is an articulation type where the tongue does not fully stop the flow of air, producing an implied note that sets the tongue up for the next note in the melodic phrase. Although articulation is just one facet of trumpet playing, it already involves the complex self-organisation of various parts of the body. These articulation options become an important part of the improvisor’s flexible and adaptive navigation of musical situations.

## Degeneracy and multifunctionality in trumpet playing

**Fig. 2:**
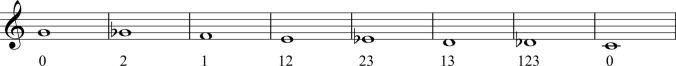
Seven chromatic valve combinations

As previously mentioned, there are seven possible conventional valve combinations. These seven combinations fit within the first interval C–G (Fig. 2).

**Fig. 3:**
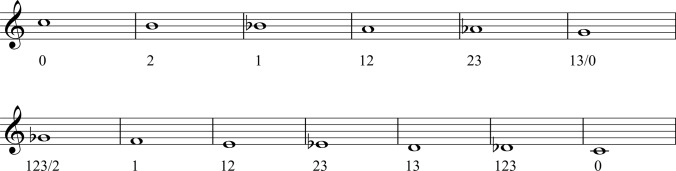
Alternative valve positions

However, as the performer ascends the harmonic series, the intervals between partials progressively shrink. Within the next interval G–C only five combinations fit. The remaining combinations overlap with the previous interval series meaning G and F# can now be played in multiple ways. The valve combinations change as the notes get higher and notes can have alternative valve positions, providing an irregular pitch workspace for the trumpet player (Fig. 3).

The different articulation types, ways of making pitch changes, and valve combinations available to the trumpeter suggest that there is not a clear “one-to-one mapping between structure and function” but instead involves degeneracy and multi-functionality (Kelso 2012, p. 907). Degeneracy refers to the capacity for different components to produce the same or similar behaviours, allowing for a variety of ways to execute performance tasks. Degeneracy can be seen in the coordination of various valve and alternative valve positions that allow the same melodic lines to be played using different valve combinations. Furthermore, a single pitch can be sounded using a number of different articulations and airspeed can be manipulated by the aperture of the embouchure, the tongue, and respiratory muscles. Complementary to degeneracy is multi-functionality where the same elements may have different functions (Kelso 2012). The tongue is a crucial feature of both articulation and partial movement, offering multiple functions. Likewise, the trumpet’s irregular pitch space means that the same valve combinations can produce different melodic lines.

The dynamic nature of improvisation requires skills that are both stable and flexible (Thelen and Smith 2006), and as we have seen, the trumpet presents the improvisor with a wide variety of interaction possibilities. Over time, through practice and performance, the improvisor begins to enact meaningful relationships with the instrument. This process of *attunement*, or resonance to meaningful information variables in the environment, allows the development of flexible performance solutions that make use of degeneracy and multi-functionality (Bruineberg et al. 2021).

## Dynamical systems theory and constraints on improvisation

Trumpeter Slater (2020) describes the term “spontaneous improvisation” as equivalent to self-organisation under a set of constraints (p. 35). Following this, I take the improviser to be an adaptive system whose behaviours are self-organised responses to a set of constraints. The tools provided by dynamical systems theory prove useful for describing this type of adaptive self-organising behaviour. For example, improvisers will have patterns of behaviour that are, depending on the circumstance, more resistant to perturbation than others. In dynamical systems theory, these stable patterns of behaviour are referred to as *attractors*. These attractors are represented on a topographical space referred to as a *phase portrait*. Areas with deep basins indicate stability and resistance to perturbation. Areas with shallower basins are more indicative of instability and less resistance to change (Strogatz 2015). These concepts can be used to describe how a trumpet player might change articulation in the course of an improvisation. For example, a slower tempo might afford single tonguing. While at faster tempos this technique becomes less stable, perhaps resulting in the use of slurring and legato back-tonguing (this is not dissimilar to the changes in gait as one transitions from a walk to a jog to a run). In other words, at moderate tempos, all articulation types are relatively stable (implying a deeper basin of attraction). But as the activity reaches a critical tempo or speed the single-tonguing attractor becomes shallower and less resistant to perturbation while the back-tonguing and slur remains stable. This prompts the system to organise toward the techniques that are more stable at this speed, namely the back-tonguing and slur. This is of course just one example of how dynamical systems theory could be used to examine improvisation (For more examples see Van Der Schyff et al. 2018; Walton et al. 2015).

## Constraints in improvisation

Constraints are certain features or structures that determine the degrees-of-freedom of a self-organising system, shaping the types of behaviours the system can perform. Constraints can enable behaviours that would not otherwise be possible and even be used to bias a system’s behaviour towards “positive functional outcomes” (Baggs et al. 2020, p. 5). In fact the term *constrain-to-afford* is a key tenet of ecological dynamics approaches to skill acquisition (Renshaw et al. 2019). This is because by shaping the kinds of actions we can perform, constraints enable different kinds of affordances to attend to.

Newell (1986) proposes that three categories of constraints interact to produce coordinated behaviour: organism (or individual), environmental, and task constraints. Individual constraints refer to the structure of the trumpet player such as posture, respiratory muscles, facial muscles, and fingers. It may also be extended to the playing experience of the individual as well as their motivation and emotional state. Environmental constraints include the physical, social, and socio-cultural environments as well as the events and objects that exist within them (Davids et al. 2007). For example, this would include the performance space (physical environment), the music generated by other performers (musical environment), the social environment (audience, or relationship with other ensemble members), and the wider sociocultural environment (community, scene). These environmental constraints are external structures that challenge the stability of the individual. To regain stability, after some perturbation from the environment, the individual must adapt or self-organise in some way dependent on a third element, the task.

Task constraints refer to the rules, intentions, or activity goals imposed on or by the individual (Newell 1986). Task constraints are ways we can choose to structure our behaviour (in a parameterised way rather than prescriptive). For example, imposing a harmonic framework to improvise within (or outside of). Improvisers might also bring to a performance a subset of tasks to move between such as attempting to synchronise with ensemble members or “pull the rug out*”* (See Ravn and Høffding 2021). Features of the environment might constitute a task constraint depending on the context. Environmental constraints reflect the ambient conditions surrounding the performer as opposed to specific tasks imposed to produce a result. The specificity of performance tasks, that is the degree to which a performance task constrains the activity, also impacts how patterns of behaviour form. This is again as the system’s ability to self-organise is contingent on its degrees-of-freedom. A common example in certain styles within the jazz genre is the need to play with certain inflections, language, or within a strict harmonic framework. These normative rules constrain the various ways in which an improviser responds to the environment.

## Musician–instrument phenomenology and dynamics

Before concluding, I would like to briefly clarify some interactional and experiential aspects of the relationship between improvisor, instrument, and environment. For expert musicians their instruments can become “transparent”–this refers to how the instrument becomes incorporated into their cognitive domain as an almost seamless extension of their body (something that the musician perceives the world with and through) (Nijs 2017; Nijs et al. 2013). In this instance the equipment or instrument functionally becomes part of the individual constraints. This type of experience can be described in a number of ways (for e.g. ready-to-hand, smooth coping, maximal grip), but generally this is seen as a result of things going well. The instrument does not interrupt the musician’s skilful engagement with the environment or *flow* (Csikszentmihalyi 1990). Even for expert improvisors this can be difficult to achieve as the dynamic musical environment offers numerous opportunities for small breakdowns that temporarily bring the instrument to the improvisor’s attention.

Furthermore, the context or task can specify the way in which the instrument presents itself or withdraws from an improvisor’s attention. Many improvising musicians explicitly centre their practice on the range of affordances made available by the instrument. The instrument becomes a focal point of the task domain, resisting withdrawal from one’s perception. From a dynamical perspective, some musicians will intentionally seek out unstable instrument interactions (also recall the previous discussion on exploring hidden affordances). For example, saxophonist Torban Snekkestad notes that at times his solo improvisation with the saxophone is more like playing duo. The instability (shallow basins) of certain interactions such as multiphonics almost gives the sense of interacting with another agent. One that can play with you or against you (Ravn and Høffding 2021, p. 531). A key point here is that our skilful engagement and phenomenological experience is highly dependent on the state of the individual, environment, and task (See Christensen et al. 2016). The improvisor’s aim may be to engage with the musical environment directly or they may put the instrument at the centre of their improvised practice. In other words, it seems that predominantly the instrument fluctuates between transparent, translucent, and opaque.

## Conclusion: some additional E’s

At the start of this paper, I suggested that we enquire into the nature of improvisation by examining some of its broader aspects, relating to the adaptive and embodied ways we interact with our environments. To capture this, I considered improvisation using principles from ecological and dynamical systems approaches. The ecological concept of affordances established that improvisers perceive emergent relations between the agent and environment or *opportunities for action*. This bidirectional relationship between agent and environment remained a theme throughout the paper. It was found that affordances can solicit different forms of actions depending on features of the physical and social environment, and the abilities and preferences of the improviser. Furthermore, it was noted that our perception of affordances is dependent upon aspects of our intention, attention, and calibration. In the second section, I examined the way an improviser engages with available affordances to navigate musical situations. I detailed the activity of trumpet playing in terms of synergetic and dynamical interactions between the physiology of the trumpeter and the material and mechanical elements of the trumpet. This view illustrated the ways that trumpet players make use of degeneracy and multi-functionality to adopt flexible task solutions. The third section expanded upon this dynamical view, seeing the improvisor as an adaptive dynamical system under a set of constraints.

While so far, I have been predominantly referring to principles from ecological psychology and dynamical systems theory, many of the ideas I have mentioned align with what is known as 4E cognitive science (Newen et al. 2018). The 4E framework seeks to provide an alternative to the still pervasive cognitivist program that focuses on information-processing in the brain. Although many of the 4E perspectives overlap, the general view is that cognition is:

1. Embodied: Cognition is not a process reserved solely for the head but is instead constituted by the body as a whole.
2. Embedded: The body does not act within a vacuum but is instead *embedded* within a rich ecological environment (physical, social, and cultural).
3. Extended: The interactions between an embodied agent and an object in the environment (such as a tool or instrument) allow us to augment or *extend* our abilities to act in ways that would otherwise be difficult or impossible.
4. Enactive: Meaning making emerges out of adaptive interactivity between an agent and their environment. By adaptively engaging with our environments, we enact meaningful experiences.

The four Es provide a valuable framework for exploring music, improvisation, and creativity (See Schyff et al. 2022; Van Der Schyff et al. 2018). For example, Torrance and Schumann (2019) draw a parallel between the enactive concept of sense-making and improvisation suggesting that the “continual unfolding of the process of an organism’s meaning making encounter with its environment is like an improvising jazz musician generating musical responses that make sense in the context of her fellow players’ (and her own) previous musical “moves” (p. 254). In the previous sections I mentioned that trumpet players utilise a repertoire of articulations and valve combinations to navigate musical environments. These, as well as timbre and pitch choices, emerge through the trumpeter’s sense-making activity. Their significance or valence are enacted as they relate to the improviser’s self-maintenance.

The use of 4E aligned frameworks shows great promise in both describing and analysing an improvisor’s practice as well as finding new avenues and tools for practitioners to improvise with. Although I draw informally from my own practice there is potential for further work that provides more personalised insight into behaviours and processes of improvisor practitioners. For example, Høffding and Snekkestad (2021) explore and categorise the improvisatory techniques used by saxophonist Torban Snekkestad. The authors focus on Snekkestad’s technical abilities, perceptual techniques, and mental and meta-techniques. Although the authors’ use of enactive-ecological terminology is somewhat minimal, it clearly underlies the research. By offering a detailed account of Snekkestad’s personal improvisatory techniques the authors provide insight into the enactive aspects of improvised performance such as the improvisor’s agency, instrument-agent relationship, and relationship to the audience. Further uses of this 4E perspective can also be seen in the field of practice-led research. For example, Slater (2020) and McLean (2018) employ ecological and embodied frameworks in their practice to develop novel improvisatory tools and skills. This paper seeks to contribute to this line of work by presenting an explicitly ecological and dynamical approach, helping to clarify how the concepts and principles from these approaches may relate to music improvisation. More specifically I have attempted to offer some preliminary suggestions at how these principles might apply in the context of a specific musical practice, namely improvised trumpet playing. It is hoped that continued enquiry will further enhance this type of research. For example, looking more closely at the types of phase transitions a trumpet player enacts within the contingencies of performance (DST), as well as how these experiences might be described using a 4E lens. These insights could potentially benefit research and practice in both music and the cognitive sciences.

## Footnotes

1 An alternative but similar effect is the multiphonic which is produced by singing and playing the trumpet simultaneously.
